# SFRT combined with immunotherapy for a growing hepatocellular carcinoma after the failure of anti-angiogenesis and anti-PD1 treatment: a case report

**DOI:** 10.3389/fonc.2025.1591424

**Published:** 2025-09-19

**Authors:** Jiamiao Hu, Yue Jin, Mengjia Wang, Yuke Pang, Shenkangle Wang, Xuyun Xie, Zhewen Wang, Xiaonan Sun

**Affiliations:** Department of Radiation Oncology, Sir Run Run Shaw Hospital, School of Medicine, Zhejiang University, Hangzhou, China

**Keywords:** HCC, SFRT, immunotherapy, case report, combination (combined) therapy

## Abstract

**Background:**

Hepatocellular carcinoma (HCC) is one of the prevalent tumors worldwide, posing a global healthcare threat. The existing treatment options for large HCC have poor therapeutic effects and are prone to drug resistance. Spatially fractionated radiation therapy (SFRT) is a highly precise radiotherapy technique that delivers a concentrated high dose of radiation to a well-defined tumor target while minimizing radiation exposure to the surrounding normal tissues. SFRT specially delivers a non-uniform radiation dose to the target area instead of a homogeneous dose throughout the tumor volume. This steep dose gradient within the targeted tumor could increase the immune-rich infiltrate within the tumor, thus enhancing the efficacy of immunotherapy.

**Case report:**

A 22-year-old man was diagnosed with large HCC, classified as Barcelona Clinic Liver Cancer (BCLC) stage C. The patient received first-line systemic treatment with bevacizumab and atezolizumab, followed by locoregional therapy with hepatic arterial infusion chemotherapy (HAIC). The tumor rapidly grew over the next 2 months. Subsequently, the patient underwent SFRT combined with anti-PD1/CTLA4 (anti-programmed death 1/anti-cytotoxic T-lymphocyte antigen-4) immunotherapy and anti-angiogenesis treatment. SFRT was administered using volumetric modulated arc therapy, delivering 26.68 Gy in two fractions every other day to the high-dose spheres and 8 Gy in two fractions to the targeted tumor. The tumor regressed nearly 40% over 2 months after the treatment, without significant treatment-related side effects (grade 3 or 4 acute and subacute toxicities) observed during the subsequent follow-up exams.

**Conclusion:**

SFRT combined with immunotherapy is a promising strategy for large HCC.

## Introduction

Hepatocellular carcinoma (HCC) is one of the most commonly diagnosed cancers worldwide and represents a major global healthcare challenge (1). Patients with HCC have a variety of treatment options, including liver transplantation, surgical resection, percutaneous ablation, and radiation, as well as transarterial and systemic therapies (1). With the development of immune checkpoint inhibitors (ICIs), anti-vascular endothelial growth factor (VEGF) monoclonal antibodies, and multi-targeted tyrosine kinase inhibitors (TKIs), the therapeutic options for advanced HCC are more diverse than ever (2). Despite continued progress in first-line treatments, the presence of immunosuppressive properties within the tumoral microenvironment and drug resistance make the treatment of HCC in the late stage extremely challenging. Therefore, the management of patients with HCC who had experienced treatment failure with immunotherapy and multi-targeted inhibitors has become an emerging issue in clinical practice. Spatially fractionated radiation therapy (SFRT) could be an effective treatment for advanced bulky liver cancer, given the bulky tumor burden and the suboptimal local control rates despite radiation therapy (RT) dose escalation. SFRT is a non-uniform dose distribution technique. It divides the treatment volume into partial sub-volumes, with high and low doses alternated among the sub-volumes. The SFRT technique includes both grid and lattice modalities, which uses image guidance techniques to localize the position and the direction of the radiation beam in order to produce alternating distributions of the high-dose (peak) and low-dose (valley) zones (3). SFRT plays an important role in the treatment of bulky primary and metastatic malignancies, demonstrating high clinical response rates and minimal toxicity (4). Among the metastatic malignancies treated with palliative intent, metastases to the lymph nodes, intra-abdominal structures, and the lung are the most common and treated by over half of responders. Among the bulky primary tumor sites treated with curative intent, head and neck cancers, primary lung cancer, soft tissue sarcomas, primary cervical cancer, osteosarcomas, liposarcomas, and melanomas prevail (5, 6). The biological basis of SFRT is not quite clear. It is hypothesized to be related to a variety of factors, including the dose–volume effects, the radiation-induced bystander effects, the microvascular effects, and immunomodulation (7–14).

In this study, we present, based on the CARE guidelines (15), the case of a 22-year-old male patient with a bulky HCC (>10 cm) who received SFRT using volume intensity modulated radiotherapy (volumetric modulated arc therapy, VMAT)-based simultaneous integrated boost (SIB) after failure of the first-line targeted and immunotherapy combined with locoregional therapy, hepatic arterial infusion chemotherapy (HAIC). Subsequent to SFRT, from September 2024, the patient continued to receive dual immunotherapy combined with targeted therapy, including cadonilimab and lenvatinib.

## Case report

### Case description

**Figure 1:**
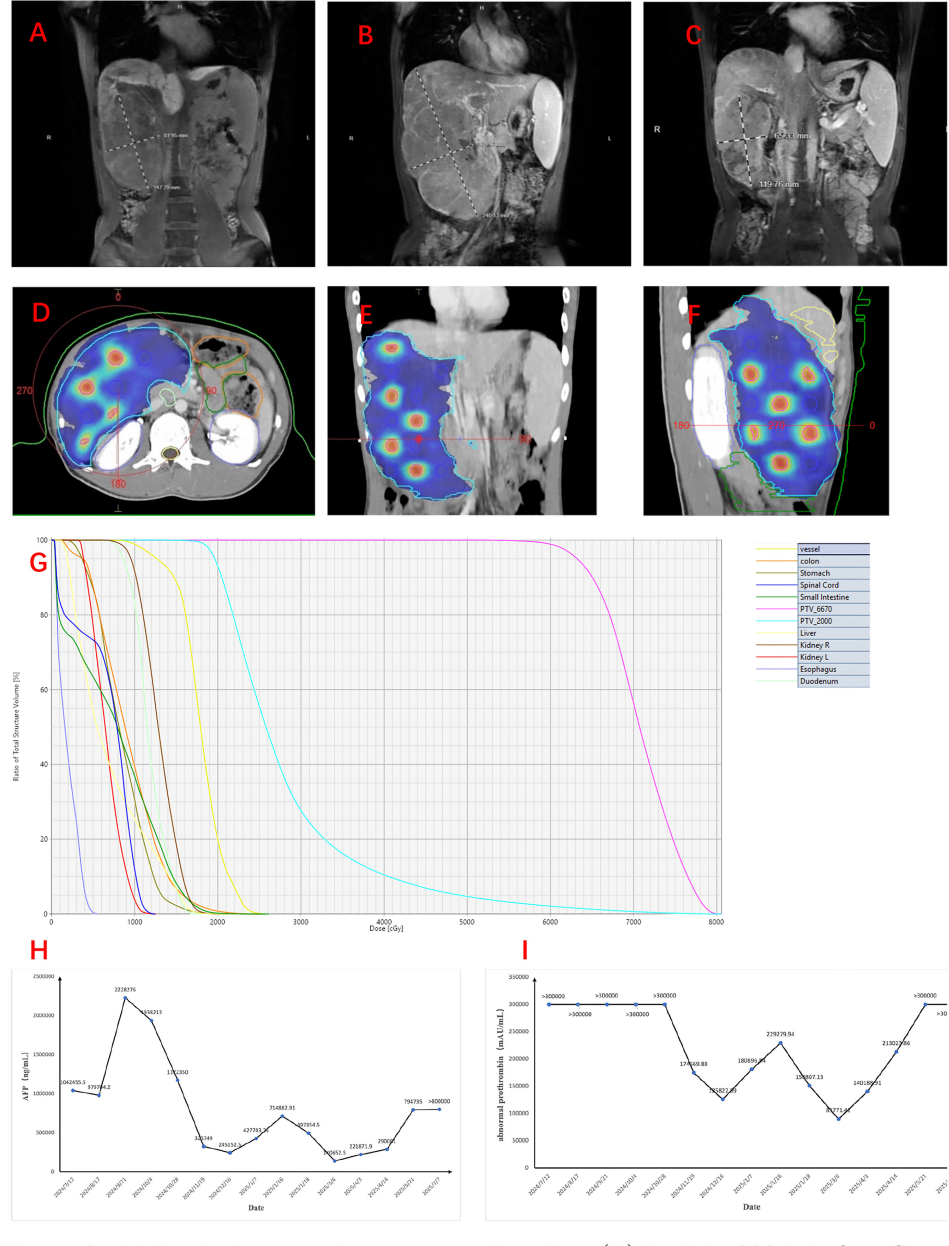
Abdominal magnetic resonance imaging. **(A)** In July 2024, before first-line treatment. **(B)** In September 2024, before spatially fractionated radiation therapy (SFRT). **(C)** In December, 2024, 1 month after SFRT. **(D–G)** Figures showing the sphere disposition in the target lesion of the hepatocellular carcinoma (HCC) patient and its associated dose–volume histogram (DVH) over the entire treatment course (five fractions). The PTV_6670 volume is delineated in *magenta*, while that of PTV_2000 is delineated in *blue*. **(H, I)** Alteration of the serum level of alpha-fetoprotein (AFP) **(H)** and abnormal prothrombin **(I)**.

A 22-year-old man, with no medical and family history, presented with an Eastern Cooperative Oncology Group performance status of 1 and preserved hepatic function (Child–Pugh class A). The patient reported progressive right lower quadrant abdominal pain beginning in July 2024. The laboratory test results showed positive hepatitis B surface antigen, elevated hepatitis B virus (HBV) DNA, and a markedly increased serum level of alpha-fetoprotein (AFP; 1,042,455.50 ng/ml), as well as abnormal prothrombin (>300,000.00 mAU/ml). A contrast-enhanced magnetic resonance imaging (CE-MRI) performed in mid-July 2024 revealed maximum axial dimensions of 12.8 cm × 7.0 cm (**Figure 1A**) at the right lobe of the liver. It also revealed cancerous thrombosis of the right main trunk of the portal vein and its multiple branches, as well as the left main trunk of the portal vein. The patient was clinically diagnosed with HCC, classified as Barcelona Clinic Liver Cancer (BCLC) stage C.

### Treatment

The patient received initial treatment, from July to August 2024, with two cycles of bevacizumab (15 mg kg^−1^ day^−1^, intravenous infusion) and atezolizumab (1,200 mg/day, intravenous infusion), followed by two cycles of RALOX-HAIC treatment, which adopted oxaliplatin (160 mg/day i.a.) and ramucirumab (4.8 mg/day i.a.). The patient was also initiated on antiviral therapy with entecavir (0.5 mg/day) since July 2024 for the management of chronic hepatitis B. After the initial treatment, a new liver CE-MRI at the end of September 2024 demonstrated maximum axial dimensions of 16.5 cm × 8.0 cm (**Figure 1B**) for the masses and nodules in the right hepatic lobe, which were larger and more numerous than before, and multiple branch thrombus formation in the main trunk and the left and right branches of the portal vein.

Imaging indicated disease progression. Lattice SFRT followed by dual immunotherapy and targeted therapy were recommended to the patient after multidisciplinary review.

The patient underwent a planning CT scan with contrast when a 4D-CT and an end-exhale breath-hold image were acquired. All visually identifiable gross disease was included in the gross tumor volume (GTV) (GTV_2000). A 0.5-cm isotropic expansion was used to create the planning target volume (PTV_2000), which was planned to receive 2,000 cGy in five fractions (EDQ2 ≈ 23.3 Gy). A total of 14 “spheres” with a diameter of 1.5 cm were delineated and regularly placed inside the GTV to create the lattice vertices inside the PTV, providing inhomogeneity within the tumor. Among them, 12 were nearly intact, while the remaining two were only minimally present. Their center-to-center distance and the successive axial planes of the spheres were 6.0 cm and 3.0 cm, respectively. The volume of these spheres was defined as PTV_6670 and was dosed at 1,334 cGy per fraction, with a total of five fractions (EQD2 ≈ 129.7 Gy) (**Figures 1D–G**). The volume of PTV_2000 was 1,639.97 cm^3^, while that of PTV_6670 was 19.64 cm^3^. Lattice stereotactic body radiation therapy (SBRT) sessions were conducted on alternate days, and a cone-beam CT (CBCT) was obtained immediately before treatment to verify tumor positioning.

In addition, any PTV_6670 vertices located within 1.5 cm of the organs at risk (OARs) were completely removed to limit the dose to normal tissue given any uncertainties at the time of treatment. The planning directives adhered to OAR constraints consistent with the five-fraction SBRT guidelines published in the American Association of Physicists in Medicine (AAPM) Task Group 101 (16). All OAR planning objectives were met (**Table 1**). The number of fractions (two to five) administered to the patient were dependent on factors such as the performance status, the blood test results, and the image sharpness of the CBCT. Eventually, the patient only completed two fractions of SFRT as originally planned from October 8 to 10, 2024.

**Table 1:** Total dose constraints over the entire treatment course (five fractions) for selected critical organs at risk.

Variable	Actual value	Dose constraints
Spinal cord	*D*_max_ = 12.51 Gy	*D*_max_ ≤ 30 Gy
Great vessels	*D*_max_ = 27.52 Gy	*D*_max_ ≤ 53 Gy
Esophagus	*D*_max_ = 4.88 Gy	*D*_max_ ≤ 35 Gy
Stomach	*D*_max_ = 22.02 Gy	*D*_max_ ≤ 32 Gy
Duodenum	*D*_max_ = 16.72 Gy	*D*_max_ ≤ 32 Gy
Jejunum and ileum	*D*_max_ = 23.35 Gy	*D*_max_ ≤ 35 Gy
Colon	*D*_max_ = 24.25 Gy	*D*_max_ ≤ 38 Gy
Liver	Total volume = 504.5 cc *V*_≤ 21 Gy_ = 504.5 cc	*V*_≤ 21 Gy_ > 700 cc
Left kidney	Total volume = 200.0 cc *V*_≤ 17.5 Gy_ = 200.0 cc	*V*_≤ 17.5 Gy_ > 200 cc
Right kidney	Total volume = 177.8 cc *V*_≤ 17.5 Gy_ = 177.5 cc	*V*_≤ 17.5 Gy_ > 200 cc

The treatment plan was implemented utilizing an Elekta Infinity linear accelerator. Upon finalization of the lattice SBRT contouring and treatment planning through the MONACO treatment planning system (version 6.0), the integrity and the deliverability of the plan were rigorously assessed in accordance with the standard clinical SBRT QA protocol, which involved reviews by both physicians and physicists.

The patient reported good tolerance of the treatment without significant adverse effects related to radiation. Subsequently, the patient received sequential dual immunotherapy combined with targeted therapy, which included cadonilimab (500 mg/day, intravenous infusion) and lenvatinib (8 mg/day, orally) for six cycles after SFRT, from September 26 to April 3, 2025. During this treatment course, the patient underwent transarterial radioembolization (TARE) with yttrium-90 (Y90) on January 16, 2025.

Imaging re-evaluation on March 7, 2025, demonstrated that some of the intrahepatic lesions had enlarged (maximum axial dimensions of 12 cm × 7 cm) and increased significantly in number, indicating disease progression. Given the patient’s concomitant hepatic decompensation (Child–Pugh class B), intensive systemic therapy was not recommended. Accordingly, the regimen was modified to two cycles of regorafenib combined with cadonilimab from April to May 2025.

**Figure 2:**
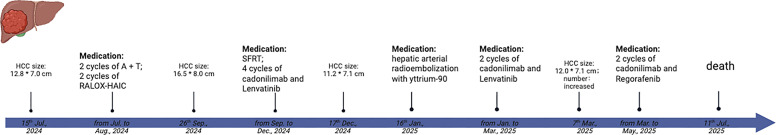
Treatment timeline of the patient with advanced bulky hepatocellular carcinoma (HCC).

The key time points of the patient’s treatment course are summarized in **Figure 2**.

### Outcomes

AFP and abnormal prothrombin (PIVKA-II) are critical biomarkers in HCC, aiding in the diagnosis, monitoring the treatment response, and predicting prognosis due to their strong correlation with tumor burden and biological aggressiveness. The serum levels of AFP and PIVKA-II decreased rapidly within 2 months after SFRT (**Figures 1H, I**). In addition, under the background of anti-PD1/CTLA4 (anti-programmed death 1/anti-cytotoxic T-lymphocyte antigen-4) and anti-angiogenesis treatment, the tumor regressed nearly 40% (11.2 cm × 7.1 cm) over 2 months after SFRT (**Figure 1C**). A re-evaluation CE-MRI on March 22, 2025, at 5 months post-SFRT showed that the intrahepatic lesions had enlarged and increased in number, indicating disease progression. Regrettably, the patient finally succumbed to the disease 9 months after SFRT (July 11, 2025). The patient’s progression-free survival (PFS) after SFRT was 5 months, and his overall survival (OS) was 1 year.

## Discussion

This case describes the adaptive management of advanced HCC in a young patient and the potential of the combination of salvage SFRT and immunotherapy in reversing tumor progression. The SFRT was designed to enable dose escalation within the GTV while limiting the dose to the surrounding OARs. We did not expect late significant side effects since the dose constraints were followed using an EQD2 dose summation despite the short follow-up.

In the landmark IMbrave150 trial, the combination therapy of atezolizumab plus bevacizumab delivered an overall response rate (ORR) of 29.8% and a 5.8-month survival benefit over sorafenib in patients with unresectable HCC (17). This trial ushers in a new era of ICI treatment for HCC. Nonetheless, patients with HCC still have poor prognosis due to tumoral heterogeneity, drug resistance, and the immunosuppressive microenvironment.

It has been proven that radiotherapy can achieve radiation-mediated immune activation through pro-inflammatory cytokines and the engagement of the innate and adaptive immunity for immunogenic tumors (18–20). SBRT has gradually become an alternative treatment option for unresectable HCC. A series of case reports demonstrated impressive tumor control from the combination of SBRT and checkpoint inhibitors, as well as anti-angiogenesis drugs, in patients with large tumors of advanced HCC (21, 22). Furthermore, another case report suggested that combining immunotherapy with bevacizumab post-SBRT could evoke an abscopal effect in a case of HCC (23).

However, both conventional and ablative radiation regimens homogeneously target the tumor with an additional margin, which leads to the decrease of the majority of circulating naive T cells at critical points of cross-presentation, as well as increasing toxicity to the surrounding normal organs (24). It is useless in anti-immune response.

SFRT, a novel radiation technique, could limit the ablative doses to tumor sub-volumes, resulting in a highly heterogeneous dose deposition within the tumor. This peak-and-valley distribution of SFRT might increase the immune-rich infiltrate within the targeted tumor, leading to enhanced antigen presentation and activated T cells (7, 25–29). The combination therapy could reprogram the immunosuppressive tumor microenvironment to making it more immunogenic and synergistically augment the antitumor response. Thus, coupling SFRT with ICIs is a reasonable promising strategy for immunogenic tumors.

It is worth noting that the combination of PD1–PDL1 inhibitors and CTLA4 inhibitors remains in the mainstream of HCC immunotherapy clinical trials. Mechanistically, PD1–PDL1 inhibition augments the antitumor activity of effector T cells, while CTLA4 inhibition can increase the infiltration of CD4^+^ and CD8^+^ T cells within the tumor (30, 31). Based on these findings, in this case, we chose an anti-PD-1/CTLA-4 antibody (cadonilimab) followed by SFRT to enhance the immune response.

In this reported case, the patient was finally administered 2,668 cGy in two fractions to PTV_6670 and 800 cGy in two fractions to PTV_2000. This fractionation scheme was determined based on the following considerations. Firstly, the uninvolved hepatic parenchyma of the patient was only 504.5 cc, and the blood test showed that the patient had abnormal liver function (AST > 10*ULN) prior to and during the course of radiotherapy. Secondly, the precision of CBCT is unsatisfactory for abdominal organs. Thirdly, the optimal SFRT dose for triggering systemic immune effects has not yet been established. Finally, our center had a relatively limited experience in the design and delivery of SFRT regimens for complex hepatic lesions, particularly in patients with compromised liver function. Thus, reducing the fraction and the dose of SFRT could be a reasonable and safe option for patients. Using MRI to guide lattice SBRT might be a promising treatment option for patients with abdominal disease.

It should be noted that we recommended that the patient complete the subsequent SFRT after observing its encouraging efficacy in December. The patient declined this therapeutic regimen and opted instead for segmental TARE with Y90. It has been reported in the literature that SBRT following TARE appeared to be effective and have acceptable tolerability (32). However, experience of combining SFRT with Y90 TARE remains limited. The treatment modality of SFRT combined with TARE requires heightened caution in subsequent clinical attempts.

This case initially adopts the SFRT technique to treat bulky HCC. Although SFRT combined with immunotherapy has shown potential for the treatment of large HCC, there is a lack of prospective multicenter clinical trials. The SFRT technology and dosimetric parameters remain diverse, and consensus on the dose prescription and technology management is required. The optimal timing and sequencing of the combination of SFRT and immunotherapy is not yet clear. Specific biomarkers for SFRT combination immunotherapy need to be further explored.

## Conclusion

Overall, SFRT combined with immunotherapy is a promising approach for the treatment of large HCC. Nevertheless, further research is required to optimize treatment regimens, reduce adverse effects, and determine the optimum combination modality.

## Data Availability

The raw data supporting the conclusions of this article will be made available by the authors, without undue reservation.
